# Development of a conceptual framework for teaching-learning of spiritual care in nursing education

**DOI:** 10.4102/curationis.v47i1.2461

**Published:** 2024-02-29

**Authors:** Ntombizodwa S.B. Linda, Hester C. Klopper, Deliwe R. Phetlhu

**Affiliations:** 1 Department of Nursing, Faculty of Science, Agriculture and Engineering, University of Zululand, Empangeni, South Africa; 2 Rectorate, Stellenbosch University, Stellenbosch, South Africa; 3 Department of Nursing Sciences, Faculty of Health Care Sciences, Sefako Makgatho Health Sciences University, Pretoria, South Africa

**Keywords:** conceptual framework, survey list, probability view, classical view, nursing education

## Abstract

**Background:**

Conceptual frameworks are not only necessary for maintaining and preserving nursing knowledge through their unique contribution, but they also assist in the organisation and provision of complex nursing interventions. The lack of formal integration of spiritual care in health professions’ education is blamed on the unavailability of guiding models among other challenges such as unavailability of relevant theories.

**Objectives:**

The objective of this article was to describe the process followed to develop a conceptual framework as the basis for a practice theory for teaching-learning of spiritual care in nursing.

**Method:**

An overall theory generative methodology was used. To develop the conceptual framework, conclusion statements deduced from empirical data using deductive and inductive strategies were applied.

**Results:**

The main concepts were identified, described, and classified. The relationship between concepts promoted synergy of the developed conceptual framework for teaching spiritual care in nursing.

**Conclusion:**

The developed conceptual framework was founded on the notion that knowledge from different sources can provide a solid base in theory generation. Therefore, the concepts of the developed conceptual framework were not only related to what is ‘ideal’; instead, their significance was underpinned by the created universal meanings for effective purposeful communication. Therefore, sources used to obtain data were critical in the development of the conceptual framework because they constituted different ways of perceiving and understanding the world.

**Contribution:**

The conceptual framework does not only guide nursing interventions but framework also provides a philosophical guide in meeting patient-centred diverse needs.

## Introduction

The scarcity of available guiding typologies, frameworks, models and theories in nursing is perceived as the overarching challenge causing the absence of formal integration of spiritual care into nursing practice. This scarcity may indicate the diminishing use of scientific-based practice founded on sound theoretical and philosophical principles. Mthembu, Wegner and Roman (2017:2) affirm the lack of guiding guidelines for spiritual care in Occupational Health Education, which potentially is suggesting the same for the other health sciences professions. Additionally, Sanders et al. (2016:214) argue against the extent and frequency of spiritual care provision that is not supported by philosophical principles of holistic care, while Neathery, Taylor and He (2020:573) warn about the potentially deceptive spiritual care that is influenced by personal spirituality and religiosity of an individual health care provider.

These existing challenges are exacerbated by the lack of conceptual frameworks and models for integrating spiritual care into nursing practice (Hoosen, Roman & Mthembu 2022:1262). In support of this notion, Nelson (2017:53) advances strong arguments in support of the sustainability of the uniqueness of the body of nursing knowledge. The same author’s argument emanates from the observed reduction in the use of conceptual frameworks and models, and states that it is a threat and potentially damaging to the body of nursing knowledge and nursing practice. Nelson (2017:53) further warns that the lack of the use of conceptual frameworks in nursing may lead to a possible decay of the nursing profession as frameworks inform the teaching and practice of nursing. Despite the importance of models in nursing, it seems that their disappearance in nursing has not been curtailed.

Burns et al. (2020:2) affirm the belief that the use of nursing frameworks must be safeguarded. Nelson (2017:53) highlights this view by saying that potentially, the loss of theories and conceptual frameworks from academic instruction is due to the fact that many practitioners in nursing regard theories and conceptual frameworks as trivial and unmeasurable. This loss of use of theories and conceptual frameworks in nursing is evident in the current trend which shows an overreliance on technology and lack of emphasis on the use of nursing philosophy. Consequently, insufficient use of frameworks may result in nursing becoming a skill-based practice instead of it being an interactive, integrative, and interpersonal practice which is critical for effective nursing outcomes. According to Burns et al. (2020), the culture of technical-based science may override the innate human instincts and/or intuition and allow nursing to be overcome by technical practice which will marginalise the caring relationship. Hence, they warn that the culture of technical-based science can negatively influence nursing practice (Burns et al. 2020:2). The interpretation of nursing practice as an art and a science implies that all aspects of human nature should be considered during care giving.

The assumption that holistic nursing cannot be achieved without embracing spirituality and/or the inclusion of spiritual care, motivated the undertaking of the current study Van Leeuwen et al. (2009). Burns et al. (2020) alert that the notion of spiritual care in nursing practice is not recognised. As a results patients are denied attention in this aspect of human need and are also deprived of integrated holistic care that they deserve. Burns et al. (2020) point to the lack of understanding by nurses about the essence of nursing as a caring practice. If nurses accept spiritual care as critical when practising nursing, educators should be concerned about how to provide spiritual guidance to students. They should equally seek objective ways to assess spiritual care competencies. Thus, Van Leeuwen et al. (2009:2865) argues that the spiritual care competency scale (SCCS) should be used to assess both practical, educational, and research-based spiritual care interventions. This scale could be used to assess spiritual competence of both students and qualified nurses. Such assessments can provide information about the areas where individual students or qualified nurses should receive spiritual guidance and/or training to become competent when providing spiritual care in clinical practice.

Therefore, we argue in this article that nurses should accept that provision of spiritual care is one of their moral obligations to patients. Reflexively speaking, to achieve holistic care nursing should embrace paying attention to all the attributes and aspects of being a human. Hence, the use of practice theories and conceptual frameworks is key to ground and guide the practice of nursing. It is based on these assertions that a conceptual framework for teaching spiritual care which aims to bridge the gap that exists in the teaching and practice of spiritual care in nursing education and practice was developed. The purpose of this article is to describe the process that was followed in developing a conceptual framework as a precursor to a practice theory for the teaching-learning of spiritual care in the undergraduate nursing programme.

Therefore, the development of the conceptual framework for teaching spiritual care in nursing was motivated by the perceived lack of guiding frameworks for the integration of spiritual care in both nursing education and practice.

## Method

This article is part of a comprehensive study that aimed to develop a practice theory for spiritual care in nursing. A theory generative methodology was used for concept identification, concept description and concept classification (Dickoff, James & Waidenbaich 1968a). The conceptual framework was developed after the empirical data collected from the nursing students and nurse educators were analysed, and specifically as a precursor to the development of the practice theory. The conclusive statements that were developed from the empirical data were analysed by laying them down on the table to identify concepts and their ‘connotative’ and ‘denotative’ meanings. The identified concepts were searched for relationship with regards to the phenomenon of spiritual care. The identified concepts were further described using dictionaries as means to establish their applicability and relationship to one another in the emergent framework. This was followed by classification process through application of the survey list of Dickoff et al. (1968a).

### Objective

Although the main objective of this article was to share the process that was followed to develop a conceptual framework as the basis for a practice theory for teaching-learning of spiritual care in nursing, it also highlights the importance of conceptual framework in nursing education and practice.

### Ethical considerations

Since the development of the conceptual framework was informed by the conclusion statements from empirical data, it is important to note that ethical approval for the study was granted with certification project registration number 13/4/22 by the University of the Western Cape Research Ethics Committee. Participants signed written consent indicating their voluntary participation in the study. Confidentiality and anonymity were observed by not revealing the names or any form of identification that would reveal their participation.

## Results

These results depict the conceptual framework development processes which include identification, description, and a classification of concepts that are central to the conceptual framework and their operationalisation using the survey list of Dickoff et al. (1968a). A significant finding of the article depicting the association between concepts and their synergic relationship is acknowledged as the unique outcome of the current study which focussed on the development of a conceptual framework.

## Process for development of a conceptual framework

Themes that emerged from data sources were refined to produce the understanding and interpretation of the findings from data sources which were constructed as conclusive statements. The conclusion statements were then analysed and produced main (core and related) concepts that formed the basis of the conceptual framework for the teaching and learning of spiritual care in an undergraduate nursing programme. Main concepts were identified, described and classified using dictionaries, and survey list respectively. The conceptualisation process started from the premise that a conceptual framework is a network of concepts which depicts their relationships and their interrelationships as interlinked counterparts within a phenomenon (Jabareen 2009:52).

According to Jabareen (2009), concepts within a conceptual framework do not only require the ‘ideal’ view about a phenomenon; but also indicate their significance, which is reflected by creating universal meaning and effective communication. This conceptual analytic process was conducted to expand their usage, applicability and functionality in the conceptual framework. In addition, Jabareen’s (2009:51) conceptual classification process was used to define concepts according to their possessed attributes as a ‘prototype’ or ‘classical’ type. The prototype view indicated concepts that were generic, with broader use and applicability, while the classical view indicated concepts that were specific, with limited use and applicability. This process was important to demarcate and position concepts in their relevant relationships in the conceptual framework. The conceptual framework development process was achieved through the creative, novel and sequential transformation of empirical data into a practice theory, which was the aim of the main study (Linda 2016:200). Conclusion statements were organised and arranged according to their nature and relationships.

## The process of identification of concepts

The process of concept identification required the arrangement of the concepts into a typology of relationships. The identified concepts were then characterised through an extensive literature description, which resulted in an emergent conceptual framework (Jabareen 2009:50). The process was embedded in an iterative mental activity in which the conceptualisations of concepts identified were further described into meaningful statements indicating ‘the nature of reality’, ‘real’ existence, and ‘real’ action. This process was aimed at depiction of both the conceptual meaning and the inherent structural functioning of the emergent conceptual framework. This was a vital step in the contextualisation of the developed conceptual framework (Dickoff, James and Waidenbaich 1968b) as applied in Nangombe and Justus (2016:47). The process of concept identification was organised through the following steps:

- ***Reading of the conclusion statements:*** This was done to identify emerging concepts from summaries of the empirical data. The empirical data was a result of the qualitative phase of the broader aim to develop a practice-theory. This process ensured logic and consistency in the extraction of the meanings of various concepts. This was followed by searching similarities of words or groups of words that represent objects, properties, or events within the phenomenon under review. This notion assisted in the process of identifying concepts which entail linguistic meaning construction through which the reality is ordered and categorised.
- ***A provisional list of potential concepts:*** This process was achieved by use of the prototype and probability view strategies as advocated by Jabareen (2009:51). Concepts were then sorted and rearranged according to their interrelatedness. Concepts were further coded according to the strength of their meanings and relatedness. Coding helped to reduce the number of concepts to a manageable size.
- ***Further review of key concepts:*** This was achieved by asking questions about the nature of the concepts and their organisation to discern their scope and ascertain which concepts are integral to the phenomenon under study. The ‘connotative meaning’ was considered to establish suggested thought, meaning, sense and intention. The ‘denotative meaning’ of concepts was also considered to establish its direct specific meaning as distinct from implied or inferred meaning.
- ***Listing of potential key and other concepts:*** Concepts were further organised by taking cognisance of concepts which were beginning to emerge as main concepts. These were coloured differently and coded. In this step ‘major concepts’ with ‘sub-concepts’ were identified. This process further revealed multiple relationships of major concepts in depicting the phenomenon under study. A revised list of main and related concepts was put through a further review to ensure rigour. The main concepts were examined for connotative meaning by checking out what sense of meaning or intention they conveyed. The dictionary was used to confirm the meanings of main and related concepts.
- ***Review of ‘denotative’ meaning:*** This was done to establish the behaviours, characteristics, actions and activities relating to the phenomena under inquiry to enhance articulation of the meaning and to reduce vagueness.

The process of identification of concepts for the emergent conceptual framework was both predictive and interpretive rather than merely descriptive. This process allowed theorisation process through which the concepts were identified, selected and organised based on their similarities (Melodie 2011:152). This process of managing the empirical data is supported by Chinn, Kramer and Sitzman (2020:801) who attest to the need to structure and contextualise a theory. Furthermore, the researcher must create conceptual meaning by identifying and defining the theoretical meaning of a concept and its importance within the conceptual framework (Chinn et al. 2020:801; Jabareen 2009:50). In the current study, this was achieved by analysing the conclusion statements that were developed in phase one of the study. Table 1 is a summary of the conclusion statements from the empirical phase, that were used to identify the concepts to develop the conceptual framework.

**TABLE 1:** Conclusion statements from empirical data.

Conclusion statements from nurse educators and clinical supervisors semi-structured interviews	Conclusion statements from nursing students focus groups	Conclusion statements from academic documents	Concepts identified
1. Spirituality refers to connectedness and is an inherent virtue in human beings that expresses a caring value. *(Concept: Caring values)*	7. Spirituality refers to connectedness to the invisible higher power and inner-self through a belief system *(Concept: Connectedness)*	13. Spiritual care relates to purposeful communication as an essential non-replaceable nursing responsibility and practice for nurses. Therefore, preparation of nursing students to become skilful communicators should accommodate a variety of spiritualties and the importance of communication that prepares the nursing students to speak clearly and listen actively as they grow personally and professionally in assertiveness and therapeutic communication with regard to patients’ culture. (*Concept: purposeful-communicators*)	Connectedness: Connected to inner-self Connected to inner emotions Connected to inner energy Connected to the invisible inborn virtue (refer to statements: 1, 7, 8, 9, 10, 12, 15)
2. Practice of spiritual care should be free from factors such as professional boundaries and, lack of role models or any unacceptable practices that are contradictory to universal ethical principles and the spirit of ubuntu. *(Concept: Ethical-based practice)*	8. Spirituality is viewed as an inborn virtue and connectedness to the inner-self through emotions driven by personal motivation and energy *(Concept: Connectedness)*.	14. The starting point of teaching-learning spiritual care should not be to impose the nurse’s spiritual, cultural and religious dispositions. Rather, it should be based on caring relationships as the means to facilitate patients’ healing and recovery from illness and state of loss of personal control. Caring interventions should be directed at assisting the patient to conduct the daily life processes for health and personhood needs which are inextricably intertwined with the patient’s cultural and spiritual beliefs. (*Concept: Belief –based intervention*)	Purposeful communicators: Importance of communication Value of communication Skilful communicators Responsible communication Accountable communicators Good character Therapeutic communication (refer to statements: 2, 4, 5, 13, 14, 15, 16)
3. Programme outcomes should integrate spiritual care throughout the curriculum and be based on good nursing philosophy and theory that caters for differences in and between different individuals’ cultures, religions and spiritualties. Outcomes should be socially acceptable to the universal law of ‘do no harm’ ‘do good’ humanitarian principle. (*Concept: Cultural-diversity*)	9. Spirituality provides a foundation for spiritual care and is connected to a complex belief system that integrates personal, religious, spiritual and cultural beliefs. *(Concept: Belief system)*	15. Teaching-learning of spiritual care should be directed at developing personal and professional responsibilities and accountability in nursing students in a manner that does not prioritise technical nursing but through promotion of caring relationships and interpersonal relationships to connect with patients while conveying to the nurse the meaning of health experience and importance of therapeutic environment. Such teaching should increase students’ awareness of own personal limitations and discovery of personal and professional values that influence execution of care in a morally sound manner. (*Concepts: professional accountability*).	Belief system: Personal beliefs Spiritual beliefs Cultural beliefs Religious beliefs (refer to statements: 3, 9, 12, 15, 16)
4. Teaching and learning of spiritual care should be facilitated in a manner that prepares students on how to instil peace in patients by reversing the de-humanising approaches to care through effective communication and relationships that promote spiritual guidance, debriefing, intuition, self-awareness, emotional health and wellbeing of patients. (*Concept: Spiritual care*)	10. Connecting with the patient at a social level is embedded in the spiritual value of caring which is demonstrated through relational and interconnectedness. (*Concept: Caring values)*	16. Nursing education should integrate caring values in a manner that promotes the patient-nurse caring-relationship not only as essential human interaction for holistic nursing but as an active ingredient in reaching out to human needs through caring-presence and purposeful relationships that cater for individuals’ beliefs, norms and values that are significant to the patient as a care receiver. (*Concept: cultural-diverse-caring*)	Cultural diversity: Cultural sensitivity Culturally acceptable care Good nursing philosophy Patient-nurse-caring relationship Caring presence Purposeful caring relationships (refer to statements: 2, 3, 9, 10, 12, 14, 15, 16)
5. Need for spiritual care is embodied in willingness, commitment, and agreement in gesture or vocalised to engage in spiritual matters where communication is regarded as talking therapy associated with the patient’s feelings of enhanced self-fulfilment, self-satisfaction and self-awareness as well as being in control. (*Concept: Purposeful communication*)	11. Teaching-learning of spiritual care should be integrated throughout the curriculum and in clinical situations with the aim of limiting perceived conflicting nursing perspectives. *(Concept: Teaching and learning)*	-	Caring values: Interconnectedness Social connectedness Inherent human virtue caring presence (refer to statements: 1, 2, 3, 4, 5, 6, 10, 12, 13, 14, 15, 16)
6. Spirituality is an inherent virtue in all human beings and is a precursor for spiritual care (*Concept: Spirituality/spiritual care*)	12. Teaching-learning of spiritual care should provide spiritual care competence to students and instil the confidence to embark on spiritual matters through the integration of caring skills, in a manner that eases and enhances connecting self with others as a means to redress the theory practice gap through appreciation of own beliefs and values, thus counteracting the lack of role models and non-conducive environment. *(Concept: Spiritual-care)*	-	Professional accountability: Spiritual care competence Moral and ethical nursing Personal & professional growth Accountable personality Unconfined spiritual care Ethically acceptable practice Ethical principle of practice Universal humanitarian principles (refer to statements: 2, 11, 13, 14, 15)
-	-	-	Teaching and learning: Curriculum integration Theory-practice gap Active teaching approaches Real life learning settings Freedom from conflicting nursing perspectives (refer to statements: 2, 4, 5, 11, 12, 13, 14, 15, 16)
-	-	-	Spiritual care: Spiritual skills Caring skills Spiritual matters Interacting or connecting with others (refer to statements: 1, 2, 3, 4, 5, 6, 9, 11, 12, 15, 16)

## The classification of concepts

After concepts had been identified as explained and depicted in Table 1, a classification using the survey list (Dickoff et al. 1968a:545–554) was followed. Classification of the core concepts enhanced their transformation into their new relational relationships by illustrating their cause-and-effect relationship. This process further explained the description of absolute and abstract relationships between concepts (Chinn et al. 2020:801). Classifying concepts further depicted how the practice theory arose from the conceptual framework. It was therefore envisaged that the use of the survey list to generate a practice theory for teaching-learning of spiritual care would guide the required competence on how to teach spiritual care and how learning should take place.

The practical steps in the development of the conceptual framework as explained by Dickoff et al. (1968a:545–554) point to three essential conditions that are required for situation producing practice theory, which are embodied in the following interventions:

- Achieving the ‘goal content’, which specifies the aim of the activity: In the current study, the goal content was the integration of a practice-theory in teaching-learning of spiritual care in the undergraduate nursing programmes.
- Determination of the prescribed activity that will make it possible to realise the goal content.
- A survey list, which in the current study, was applied to classify the conceptual-based activities and elements that would be directed towards the attainment of the goal content, which also enhanced the functionality aspect of the conceptual framework. The activities and elements related to this integration of teaching-learning of spiritual care which were derived from empirical data informed the conceptual framework by using the survey list of Dickoff et al. (1968a:545–554). The survey list assisted in classification of core concepts, it also influenced the management of data which entailed the following:
  ■ Observation of the activity’s clustering of factors, elements, and aspects relevant to producing a practice situation at the conceptual level, whether complex or simple.
  ■ Use of literature to appraise the concepts as a necessary step for achieving the overall aim of producing a practice model.

The use of the survey list further allowed the researcher to operationalise the situation producing theory’s value and its adaptability to clinical and practical nursing environments by responding to the following six questions which Nangombe and Justus (2016:47) referred to as hierarchical representation of the reasoning map:

- Who or what performs the activity?
- Who or what benefits from the activity?
- In what environment is the activity performed?
- What is the targeted outcome of the activity?
- What is the activity’s guiding procedure, protocol, or technique?
- What is the energy source for the activity?

Table 2 presents how Dickoff et al.’s (1968a:545–554) survey list was applied to the core concepts of the developed conceptual framework and ultimately to the practice theory.

**TABLE 2:** Summary of the application of the survey list.

Six survey list questions	Survey list	Application
1. Who or what performs the activity?	Agent 1	Nurse educator
	Agent 2	Clinical supervisor
2. Who or what is the recipient of the activity?	Recipient	Nursing student
3. In what context is the activity performed	Context	Nursing education
4. What is the endpoint of the activity?	Purpose	Integration of spiritual care in the undergraduate nursing programme
5. What is the guiding procedure?	Process	Teaching-learning
6. What is the energy source of the activity?	Dynamics	Commitment

Consequently, the usage of the survey list was operated as the primary step of assigning main concepts to their functional value in the conceptual framework. Furthermore, the survey list enhanced designation of their ‘nature’ and ‘role’ in the conceptual framework. For instance, the nurse educator’s role as ‘the agent’ clearly demarcating the connotative and denotative of being the facilitator in the learning activity. Similarly, all concepts were assigned to a specific role while depicting their function in the conceptual framework. Through this process of assigning concepts using the survey list, the developed conceptual framework was thus activated. After this step of assigning main core concepts in the survey list, all other concepts were then attended to. All concepts were placed in relation to each other, showing how each concept interacted with the concepts designated in the survey list. Concept interactions included actual and imagined relationships and connections.

**FIGURE 1:**
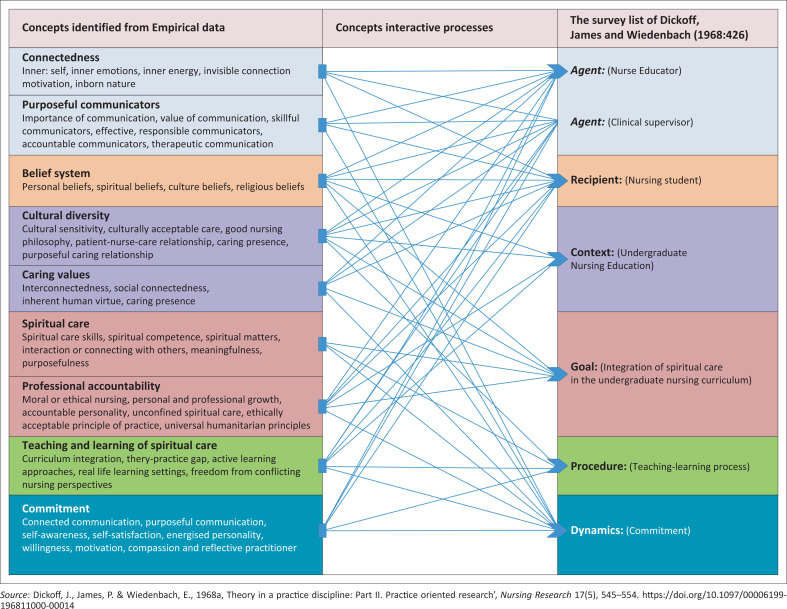
Classification of concepts using the survey list.

Figure 1 presents the picture of how the Dickoff et al. (1968a 545–554) survey list was used, to classify core concepts, and to operationalise them according to their functionality in developing the conceptual framework.

## Discussion

The main study explored the understanding of nurse educators in teaching-learning of spiritual care and reported on the existing lack of a guiding theory in the formal preparation of pre-service nurses in South Africa. It confirmed the need for a philosophical basis to support formal integration and implementation of spiritual care in nursing education (Linda, Phetlhu & Klopper 2019:2). This observation was also made by Kevern (2017:3–4), who not only raised the bar in the discourse about lack of theoretical and conceptual models for spiritual care, but who also argued against the philosophical jargon where spiritual care is viewed through the lens of psychology.

Kevern’s (2017:3) argument initiates another debate which could be one of the most critical dialogues required to achieve a common philosophical understanding of spiritual care, particularly in the Western health care system. This could be done by refocusing complex cultural beliefs, which are often misjudged and misplaced in the context of Western medicine. Kerven’s (2017) argument could be worth noting since it could discover one correct part of the puzzle. Therefore, such philosophical explanations should be provided with a deliberate goal to propel the efforts towards achieving the scientific basis of models. Such explanations will cover up the potential lack which otherwise lead to various barriers against the universal acceptance of integration of spiritual care in Western health care. Therefore, the authors of the current study are not merely joining the on-going debates on lack of universal understanding within the conversions of Western health care provision but their intention is to make a significant and meaningful contribution to an area that is critical for patient survival when faced with catastrophic life events such as irreversible diseases.

In contrast to the psychological view which denotes spirituality as an expression of one’s internal motives and desires with focus on self in search of meaning and purpose in life rather than focus on supreme intelligence (Regos et al. 2022), we the authors of the current article are of the view that human beings are not only biological beings but are complex beings who constantly navigate the continuum of ‘being human and human beings’ through investigation of self and one’s environment to discover one’s self in relation to the significant other. However, their reflection, introspection and self-investigation and discovery of who they are could be severely tempered and compromised in sickness, especially when one is undergoing a stressful experience (Rego et al. 2022). In addition, this self-deficit could exacerbate the experience of illness because of lack of innate strength to overcome the negative predicament (Van Leeuwen 2008). Therefore, spiritual care in nursing could be the crucial distinction between recovering from a devastating illness and death. Thus, it should be practised, providing the bridge between the patient’s now-reality and his or her expected-reality while going through a stressful life event such as critical illness. On the contrary, Zhang et al. (2022:3) argue about the lack of conceptual models and theories among other barriers, which should guide the development of behavioural intervention technologies (BIT) among vulnerable populations.

Therefore, we are affirming that spiritual care in nursing should be founded on the premise that it can be provided in line with its intuitive, interpersonal, selfless and integrative expressions that must be embedded in the nurse’s awareness of the transcendent dimension of life that reflects the patient’s reality. However, not all nurses can go through this journey with their patients, owing to spiritual care not being overtly and visibly practised in Western health care settings. It is thus suggested that spiritual care should be formally integrated into nursing practice and nursing curricula (Linda et al. 2019:5; Mthembu et al. 2017:2).

Therefore, the teaching of spiritual care in nursing which is intended to address patient-centred nursing interventions as a holistic and comprehensive approach, is advised. This idea of ‘wholeness’ in health care is supported by Rego et al. (2022:484) who promote the use of a biopsychosocial-spiritual model in palliative care as means to address the totality of the patients’ relational existence to the physical, psychological, social and spiritual dimensions. This scientific value of Rego et al.’s (2022) model supports the usefulness and applicability of conceptual framework that was developed in the current study as is discussed below.

## Scientific merits of conceptual frameworks in nursing education and practice

A conceptual framework for nursing provides a structure for reflection, observation and interpretation of phenomena and, specifically, it provides guidelines and guidance for aspects of clinical practice. It also provides a particular and distinct frame of reference through which nurses and patients can better manage their environment while giving and receiving health care respectively. Although numerous conceptual models exist, fewer attempts have been made to address the phenomenon of spiritual care in nursing practice in South Africa. Generally, existing models emphasise acknowledgement of four central concepts for nursing practice, which are person, environment, health and nursing, without necessarily providing a stepwise application during nursing interventions. The developed conceptual framework provides practical structure that enhances its applicability through the survey list (Nangombe & Justus 2016:47).

### Role, benefit and significance of the developed conceptual framework

The conceptual framework described here will assist nurse educators and nursing students to engage meaningfully with spirituality and spiritual care in nursing. The use of models of holistic care can promote the integration of spiritual care where all the components of being human are addressed and taken care of. The conceptual framework should be integrated into the teaching and learning of spiritual care, also taking into consideration the theoretical and technical components that constitute nursing as both a science and an art.

## Recommendations

It is advisable that nurse educators and practitioners in particular should prioritise the use of conceptual frameworks in nursing education and the practice of nursing respectively. This is supported by Hoosen et al. (2022:1278) who suggest that guidelines for teaching spiritual care and spirituality are necessary if a patient is going to remain at the centre of caring. The formal use of a conceptual framework is thus recommended because it will promote intentionality and visibility in the teaching and practice of spiritual care in the nursing profession.

The redress of the unintentional exclusion of formal integration of spiritual care into nursing is fundamental in reversing ineffective nursing care. The developed conceptual framework for teaching-learning of spiritual care in nursing provides the foundation on which the generated practice theory is grounded. It also complements the holistic nursing care approach which otherwise appeared difficult to achieve. Therefore, while the conceptual framework may not fully close the theoretical gap caused by the lack of theory to guide teaching-learning of spiritual care, it is believed that formal integration of spiritual care in nursing will expand the understanding of human suffering through illness during hospitalisation which will benefit both patient and nurse as they will have a common understanding guided by practice theory as advocated by Hoosen et al. (2022), Mthembu et al. (2017:2); Linda et al. (2019:5).
